# Cell size sensing—a one-dimensional solution for a three-dimensional problem?

**DOI:** 10.1186/s12915-019-0655-3

**Published:** 2019-04-29

**Authors:** Ida Rishal, Mike Fainzilber

**Affiliations:** Department of Biomolecular Sciences, Weizmann Institute of Science, 76100 Rehovot, Israel

## Abstract

Individual cell types have characteristic sizes, suggesting that size sensing mechanisms may coordinate transcription, translation, and metabolism with cell growth rates. Two types of size-sensing mechanisms have been proposed: spatial sensing of the location or dimensions of a signal, subcellular structure or organelle; or titration-based sensing of the intracellular concentrations of key regulators. Here we propose that size sensing in animal cells combines both titration and spatial sensing elements in a dynamic mechanism whereby microtubule motor-dependent localization of RNA encoding importin β1 and mTOR, coupled with regulated local protein synthesis, enable cytoskeleton length sensing for cell growth regulation.

## Linking genome expression and cell dimensions by cell size sensing

**Fig. 1:**
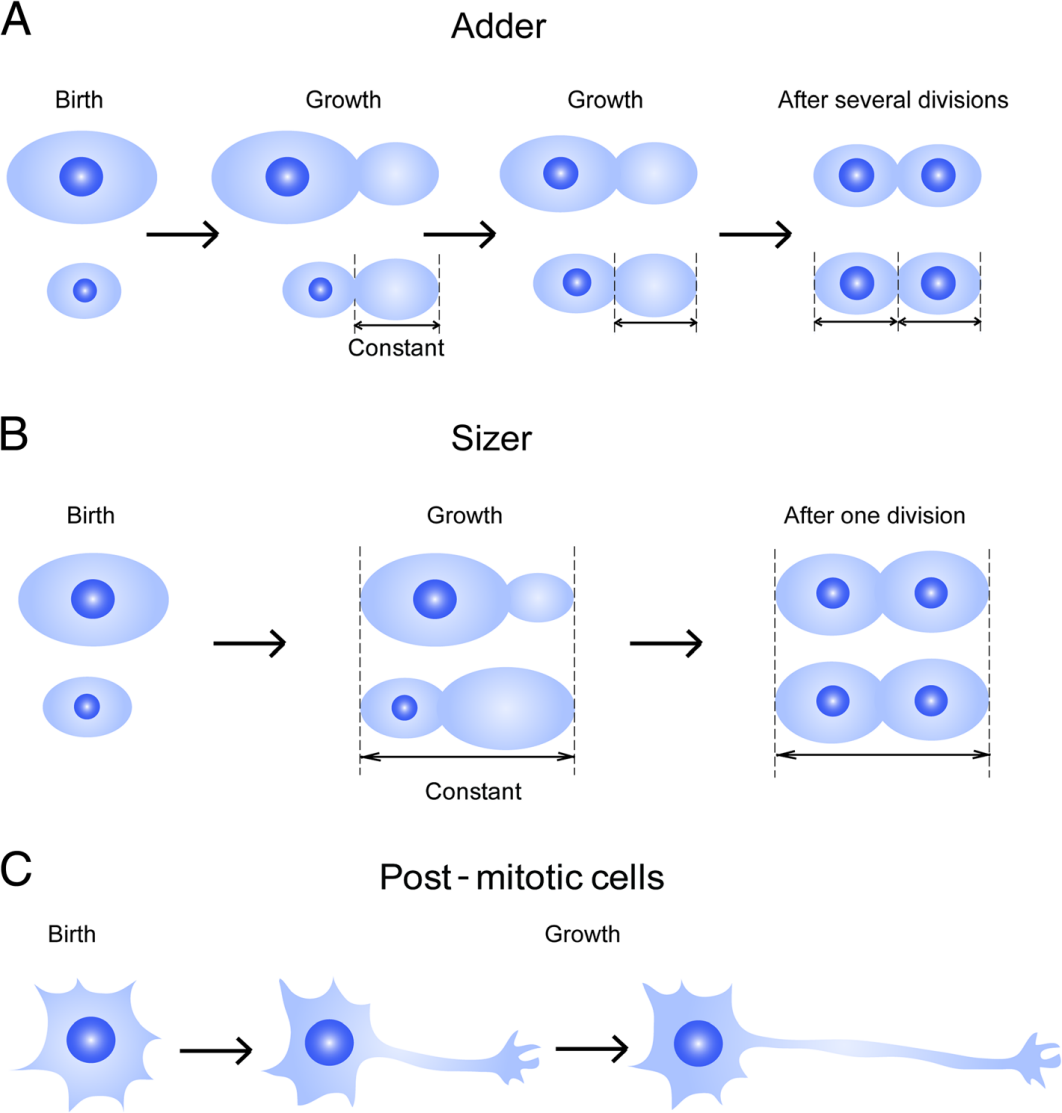
Different models for cell size regulation. **a** The adder model enables size homeostasis without active size sensing. If large and small cells add a constant amount of cell mass in each division cycle, size variations will be reduced over multiple divisions to reach a uniform cell size in the population. **b** The sizer model postulates active size sensing, ensuring that cell division occurs only upon reaching a constant overall cell mass, hence maintaining size homeostasis in each cell cycle. **c** Post-mitotic cells such as neurons grow to characteristic size ranges after birth, without any subsequent cell division; hence, their growth must be constrained by sizer-like mechanisms or by extrinsic factors

Size is a fairly uniform characteristic for any given cell type, and the reasons for this deceptively trivial observation have been vexing science since deep in the previous millennium [1]. Size control requires coordination of cell division with growth and cell cycle progression, and can in principle be regulated by a timer mechanism which assesses how long cells spend in a given stage of the cell cycle, or a mechanism which regulates growth in proportion to size, or stops growth at a specific target size [2]. Two types of models have been proposed for the latter type of mechanism (Fig. 1). The first, commonly termed the adder model, postulates that cells of different sizes add a constant amount of material before each division [3]. Under this mechanism fluctuations in size are not corrected within a single division cycle, but rather converge to a steady state size over multiple division cycles. The second sizer model postulates growth cessation or division upon attainment of a size threshold [3]. While adder or timer models could conceivably exist independently of any need for a size sensing capacity in the cell, the sizer mechanism requires such a capacity. Experiments in a variety of unicellular organisms have shown that different size regulation mechanisms may be utilized by the same cell at different stages of the life cycle [4, 5], and that adder-like phenomena may arise from sizer mechanisms operating at two distinct stages of the cell cycle [6]. Different types of mechanisms may be appropriate for different cell types; for example, adder-type mechanisms appear to be utilized by different types of microorganisms [3], including an archeal species [7]. In contrast, the requirement for multiple division cycles to correct cell size errors in the adder model renders it unsuitable for size regulation in post-mitotic cells such as neurons (Fig. 1).

Early work in yeast and animal cells provided evidence for size sensing, with observations of non-linear growth rates and size-dependent fluctuations in growth duration between division points [8, 9]. However, these characteristics are not shared by all cell types studied to date; for example, analyses of proliferating rat Schwann cells suggested that they do not require a cell size checkpoint to maintain size [10]. More recent studies on mammalian cell lines revealed a two-tier size homeostasis mechanism incorporating a size checkpoint with adder-like growth behavior [11]. Mathematical modeling of size homeostasis behavior in single-cell datasets suggested that mammalian cells operate using a near-adder mode of size control, by combining modulation of both cell growth rate and cell-cycle progression [12]. Indeed, another study using cell lines demonstrated longer growth times for smaller cells and adjustment of growth rates by larger cells before division [13]. These findings, together with additional studies showing size dependence of transcription [14], protein synthesis [15, 16] or stabilization [17], and metabolism [18], suggest that size is likely sensed in eukaryotic cells while remaining enigmatic on the molecular details thereof. The likelihood of size-sensing mechanisms in animal cells is further highlighted by drastic phenotypes observed upon size disruption in mammalian neurons [19–21] and by reports proposing evolutionary links between metabolic activity and cell size [22, 23].

## Size sensing—spatial versus titration models

Despite accumulating evidence for size sensing capability in different cell types, the molecular details of such a mechanism are not well understood. Yeast cells have been most intensively studied in this regard, and two classes of size-sensing models have been proposed—titration-based measurements versus spatial sensing. Titration-based mechanisms postulate that increases or decreases in levels of a key signal provide a critical checkpoint size signal. A recent study in fission yeast demonstrated size-dependent expression of the mitotic activator Cdc25, and suggested that size-dependent increases in Cdc25 levels trigger cell division upon reaching a threshold concentration [24]. An alternative mechanism based on work in budding yeast proposed that size-dependent reductions in concentration of the cell cycle inhibitor Whi5 is a key size regulator [16]. Reconciliation of such apparent opposites might be achieved by combinatorial titration of multiple activator and inhibitor molecules whose levels are affected differentially by cell size [25]. In this context, size might also be encoded by posttranslational or signaling modifications of the active molecules rather than absolute changes in their expression levels, as shown by a recent study linking p38 MAPK activity to size regulation in mammalian cell lines [26].

In the second class of models, subcellular localization of key signals provides size readouts to the cell. For example, in fission yeast the proteins Pom1 and Cdr2 have been proposed as components of such a mechanism, wherein Pom1 is transported to cell tips and diffuses from there to form longitudinal gradients along the cell, while Cdr2 is localized to large immobile structures at the plasma membrane in the cell middle, termed cortical nodes [8]. Conflicting findings suggested that cell size was sensed either as length encoded by a linear Pom1 gradient [27, 28] or by cell surface area encoded by Cdr2p nodal concentration [29]. More recent work has, however, suggested that cell size homeostasis is still preserved in Pom1 deletion mutants [30] and that Cdr nodal regulation is reinforced by additional components localizing in bursts to the nodes [31]. Another very recent study suggests the existence of both Cdr2-dependent and Cdr2-independent size-sensing mechanisms in fission yeast [32]. Thus, multiple levels of regulation and redundancy are likely to exist in size-sensing mechanisms, complicating elucidation of their key principles.

Other types of spatial measurements might also provide proxies for size sensing, such as monitoring the sizes of key organelles within a cell. Nuclear size is the most well-studied example, and the karyoplasmic ratio describes the tight nuclear/cytoplasm size relationship in almost any cycling cell type [33]. Intriguingly, both nucleoplasm and cytoplasm harbor membrane-free structures and organelles that scale with cell size [34]. Nucleolus size was shown to be linked to cell size by intracellular phase transitions driven by concentration changes upon successive cell divisions [35], and recent work in *Caenorhabditis elegans* intestine demonstrated a direct proportionality of nucleolus size to both cell and whole-body size throughout worm development [36]. Centrosome size and microtubule cytoskeleton dimensions provide similar examples in the cytoplasm [37–39]; thus, for example, scaling of microtubule growth rates with cell size adapts mitotic spindle length to cell volume [40].

## A length-sensing model—frequency-encoded sensing of a single dimension

Correlations of overall microtubule cytoskeleton dimensions with cell size raises the possibility of using cytoskeleton length as a proxy measurement for size sensing, thus simplifying the three-dimensional challenge of size sensing to the single dimension of length measurement [41]. Microtubules might be particularly appropriate for such measurements due to their spatial organization connecting the microtubule organizing center near the nucleus and cell center with the cortical region adjacent to the plasma membrane. Indeed, microtubule-associated transport has been implicated in length control of cilia or flagella, which are short linear projections extending a few microns from cell surfaces [42]. A model based on retrograde diffusion of the microtubule motor kinesin after delivery of its cargo by anterograde transport suggested that it could act as a length sensor within flagella [43]. A conceptually similar mechanism was previously proposed for length sensing during neuronal polarization, wherein anterograde transport and retrograde diffusion of an axon growth regulator accounts for its neurite length-dependent accumulation [44]. Although such mechanisms might function well for organelles or small cells, the range limits of intracellular diffusion gradients likely restrict their applicability in large cells [41, 45].

We looked into the possibility that active transport by the microtubule motors dynein and kinesin coordinates length sensing, using neuronal axon length as a model system. Axons comprise the largest compartment of a neuron; hence, axon length provides a proxy for overall neuronal size. Moreover, the distributed morphologies and large sizes of neurons can be advantageous in studies of compartmentalized signaling and size sensing [46]. Dynein and kinesin are inherently limited to unidirectional movement along microtubules, with characteristic velocities and transport capacity [47–49]. These characteristics provided useful constraints for modeling different configurations for motor-based length sensing [50, 51]. Simple models estimating length from signal spread or from duration of signal travel on a single motor type (the so-called “time of flight” model) were found to be unlikely by computational simulations due to noise effects and lack of robustness in the system [50]. In contrast, simulations of a bidirectional motor model revealed length-correlated retrograde oscillating signals for configurations wherein a kinesin anterograde signal stimulates a dynein retrograde signal, which then in turn represses the anterograde signal [51]. Oscillatory signals can be significantly more robust than amplitude-encoded signals [52, 53]; hence, encoding spatial information by signal frequency rather than signal quantity may be advantageous. The original simulations envisaged decoding of the oscillatory signal by biochemical or transcriptional networks in the cell [54], but a very recent modeling study suggested that this might also be done by spectral decomposition of the oscillatory signal [55]. Calculations based on available measurements of velocities for molecular motors indicate that the model would be most appropriate for a range from tens of micrometers to a few millimeters [41], fitting the sizes of most animal cell types and embryonic neurons, but not small microorganisms or adult neurons in vivo in large mammals.

An experimental test of motor-dependent oscillatory signaling for axonal length sensing was suggested by simulations showing that reducing levels of either kinesin or dynein should slow frequency decay of the retrograde signal [51]. If neuronal growth rates are proportional to retrograde signal frequency, or if growth stops when the system reaches a limiting frequency, the model predicts that reducing motor levels within a prescribed range should lead to increases in axon length [51]. Indeed, a knockdown screen in sensory neurons revealed axon lengthening phenotypes upon reduced expression of dynein heavy chain 1 (Dync1h1) or a number of kinesin heavy chains. The heavy chains are the ATP-binding subunits of molecular motors and are indispensable to their function. Further analyses in a mouse line with a point mutation in Dync1h1 revealed increased axon lengths for both adult sensory neurons in culture and embryonic sensory axons in vivo [51]. Moreover, cultured fibroblast cells from the mutant mouse also revealed size increases, suggesting that motor-based size sensing might also function in non-neuronal cells [51].

## Motor-dependent RNA localization in cell growth regulation

A follow-up study then examined a number of mouse mutants for axon-lengthening phenotypes similar to those observed upon microtubule motor knockdown, and identified such a phenotype in a mouse with an importin β1 3′ UTR deletion [56]. Both adult sensory neurons in culture and embryonic sensory neurons in vivo revealed significantly more axon growth for the importin β1 3′ UTR deletion than wild-type controls [57]. Since the main effect of this mutation is loss of importin β1 mRNA transport to axons, structure–function analyses were employed to identify the precise axon-localizing motif, which was then used to identify nucleolin as an RNA-binding protein (RBP) for importin β1 mRNA [57]. Nucleolin is a multifunctional RBP that is a major component of the nucleolus, but is also found in the plasma membrane [58, 59]; hence, it is well-placed to function in a mechanism based on sensing distance between cell center and periphery.

Disruption of the interaction between nucleolin and kinesin using AS1411, a nucleolin-targeted DNA aptamer, sequestered nucleolin from sensory axons and caused robust increases in axon growth [57]. Similar findings were obtained in 3 T3 fibroblast cells, where we found importin β1 mRNA associated with kinesin and nucleolin, and importin β1 protein associated with dynein. Strikingly, AS1411 treatment of 3 T3 cells caused a significant size increase at all stages of the cell cycle [57]. Moreover, aptamer treatment also induced a significant reduction in local protein synthesis at axon tips of cultured neurons and in the cortical domains of fibroblast cells [57]. A similar reduction in protein synthesis at axon tips was also observed in cultures of importin β1 3′ UTR deletion neurons [57]. Hence, perturbation of the subcellular localization of nucleolin or of its cargo importin β1 mRNA affects axon length or cell size. In this context, it is interesting to note that subcellular partitioning of importin α to the plasma membrane was very recently suggested to scale nucleus and mitotic spindle size to cell size [60]. It will be interesting to explore the relationship between membrane association and motor-driven cytoplasmic transport of importins in size regulation mechanisms.

**Fig. 2:**
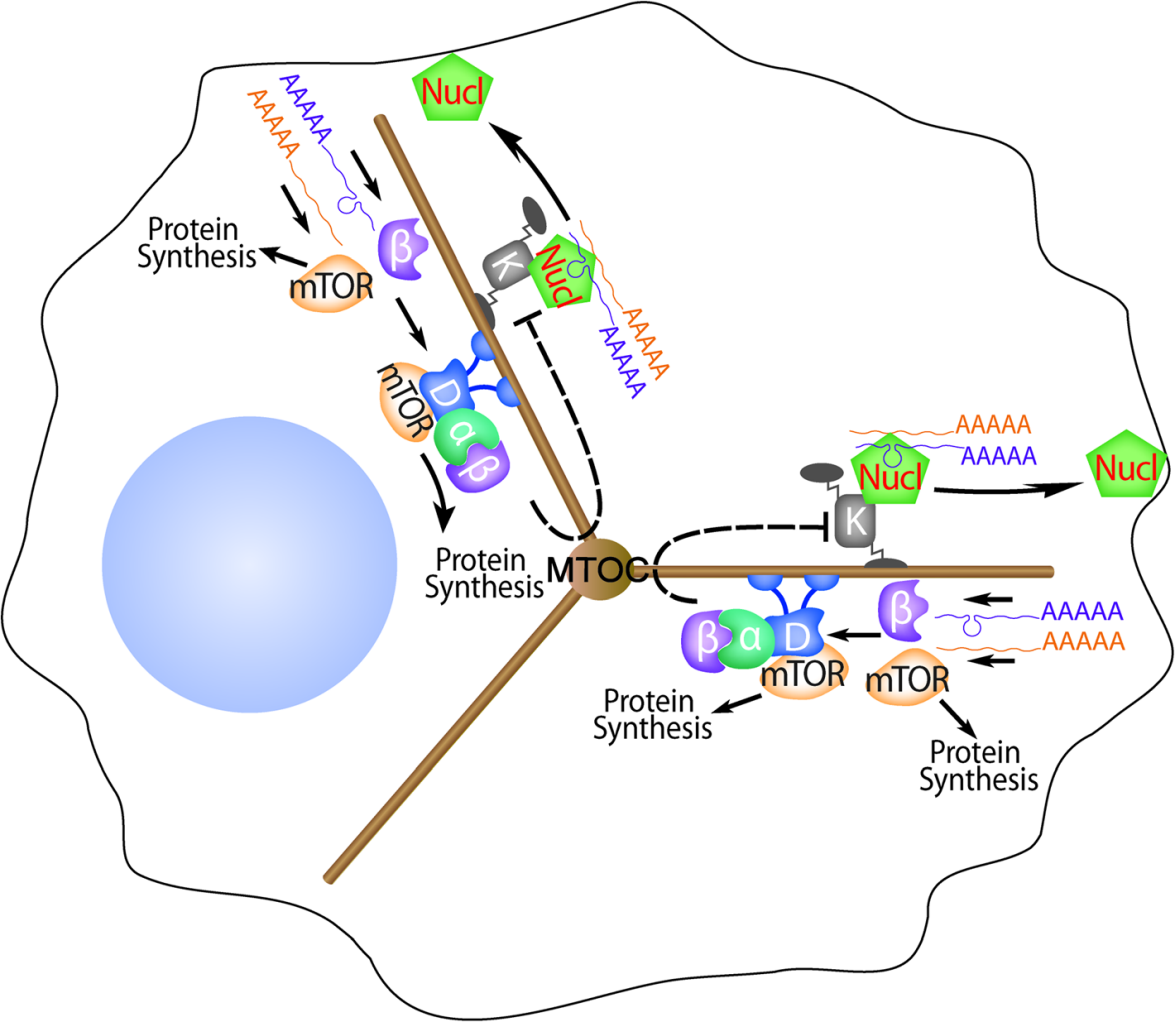
A proposed size-sensing mechanism based on microtubule motors and local translation. Kinesin motors (*K*) transport mRNAs associated with the RNA binding protein nucleolin (*Nucl*) from the microtubule organizing center (*MOC*) to the periphery of the cell. Upon arrival at the cell cortex, nucleolin-associated mRNAs undergo local translation. Localized synthesis of importin β1 (*β*), mTOR, and other proteins enables formation of a retrogradely transported complex with an importin α bound to dynein (*D*). Restriction of the complex to the cell center shifts protein synthesis locales from the periphery to the center of the cell [57]. Computational modeling of this system, incorporating a still hypothetical negative feedback loop at the cell center (*dashed lines*), suggests that it generates a fluctuating retrograde signal, the frequency of which changes with cell length or size [51]. Definitive support for this model will require elucidating the nature of the negative feedback loop and determining how the frequency encoded signal affects biosynthesis and metabolism to regulate cell size

Perturbation of nucleolin or importin β1 localization shifts the balance of protein synthesis between the periphery and the center of the cell [57]. This finding was intriguing since others have suggested that size sensing in yeast might be based on scaling of specific protein synthesis rates with size [25]. We therefore proceeded to ask how localized protein synthesis might be linked with size modulation. mTOR is a key regulator of protein synthesis, both directly through control of translation [61] and indirectly through regulation of ribosome biogenesis [62] and cytoplasmic ribosome levels [63]. Moreover, it has well-established roles in cell size and growth control [64, 65], and its multiple functions are dependent on intracellular localization [66, 67]. Hence, we examined whether mTOR regulates or is regulated by local translation. Strikingly, mTOR mRNA localizes to sensory axons in complex with nucleolin and kinesin [68]. The AS1411 aptamer reduced mTOR mRNA levels in axons while increasing them in neuronal cell bodies, further confirming that mTOR mRNA is transported in complex with nucleolin. Proteomic analyses revealed that mTOR controls most local protein synthesis in axons, including its own synthesis and that of importin β1, indicating that mTOR mRNA localization is a necessary prerequisite for localized upregulation of protein synthesis upon need [68]. Accordingly, sequestration of mTOR mRNA from axons by mutation of the mTOR 3′ UTR or by aptamer treatment reduced axonal local protein synthesis. Others have shown association of mTOR with dynein [69]; hence, taken together these findings suggest that nucleolin-mediated localization of mTOR mRNA and dynein-mediated transport of mTOR protein may enable subcellular regulation of protein synthesis to impact on cell size sensing (Fig. 2).

The text above, summarized in Fig. 2, outlines a hypothesis for cell size sensing that still requires extensive testing on multiple levels. Attractive features of this hypothesis include simplifying the three-dimensional challenge of cell size sensing to scanning the single dimension of cytoskeletal length, increased robustness due to frequency encoding rather than amplitude encoding of size signals, and the combination of features of both spatial and titration-based modes of size sensing. Indeed, one might envisage an evolutionary continuum in the development of such mechanisms. Purely titration-based sensing of key protein concentrations might have provided size readouts in early and small morphologically simple cells where diffusion ensured uniformity of the readout throughout the cell. As cells evolved to become larger and morphologically complex, protein levels could become differentially regulated in subcellular compartments, driving addition of spatial specifications to the initial titration-based size sensing mechanism. Different cell types may have evolved to utilize various combinations of these principles to fit their specific morphological constraints in size sensing. Motor-driven RNA-based localization of protein synthesis regulators allows differential regulation of biosynthesis in different cellular compartments, potentially combining titration and spatial sensing elements into one combined mechanism for large morphologically complex cells. Further characterization of the mechanism will require identification of feedback components in the system and determining how localized changes in protein synthesis can provide size readouts to cells.
